# Mathematical expertise: the role of domain-specific knowledge for memory and creativity

**DOI:** 10.1038/s41598-023-39309-w

**Published:** 2023-08-02

**Authors:** Michaela A. Meier, Franz Gross, Stephan E. Vogel, Roland H. Grabner

**Affiliations:** University of Graz, Universitätsplatz 2, 8010 Graz, Austria

**Keywords:** Psychology, Human behaviour

## Abstract

In contrast to traditional expertise domains like chess and music, very little is known about the cognitive mechanisms in broader, more education-oriented domains like mathematics. This is particularly true for the role of mathematical experts’ knowledge for domain-specific information processing in memory as well as for domain-specific and domain-general creativity. In the present work, we compared 115 experts in mathematics with 109 gender, age, and educational level matched novices in their performance in (a) a newly developed mathematical memory task requiring encoding and recall of structured and unstructured information and (b) tasks drawing either on mathematical or on domain-general creativity. Consistent with other expertise domains, experts in mathematics (compared to novices) showed superior short-term memory capacity for complex domain-specific material when presented in a structured, meaningful way. Further, experts exhibited higher mathematical creativity than novices, but did not differ from them in their domain-general creativity. Both lines of findings demonstrate the importance of experts’ knowledge base in processing domain-specific material and provide new insights into the characteristics of mathematical expertise.

## Introduction

Research on the psychological correlates of expert performance has substantially improved our understanding of the foundations and development of exceptional cognitive performance^1–3^. Numerous studies have revealed that experts, defined as individuals who show “consistently superior performance on a specified set of representative tasks for a domain” (p. 277)^4^, possess a large and well-organized domain-specific knowledge base that was acquired over several years of intensive (deliberate) practice. This knowledge base allows them to process information in their domain more effectively^1^. Individual differences in domain-general abilities (e.g., intelligence and working memory) also play important but subordinate roles for expertise^5^. Most of these insights come from studies in so-called traditional expertise domains such as chess and music, which require very specific training^6,7^. Much less is known about expertise in core academic subjects such as mathematics.

In contrast to traditional expertise domains that offer a reliable indicator of skill level (e.g., the Elo score in chess, which indicates the relative skill level compared to other players^8,9^), in mathematics, experts are typically defined as individuals who study or have studied mathematics at university level. Consequently, novices represent individuals without tertiary education in mathematics. The majority of previous studies comparing both groups focused on selected domain-general and domain-specific abilities and reported that experts perform better in intelligence, working memory, and some basic numerical tasks such as number comparison and mental arithmetic^10^. When experts and novices were matched for intelligence, only differences in some domain-specific tasks (mental representation of numerical magnitude, continuing sequences, and arithmetic fact knowledge) were observed^11^. A central limitation of this research lies in the administration of rather basic numerical tasks such as number comparison or arithmetic. Even though these tasks also draw on mathematical knowledge, they cannot capture the actual impact of the experts’ broad knowledge base on information processing.

In traditional expertise domains, the relevance of experts’ domain-specific knowledge for information processing was first observed and has been mainly investigated using a memory task originally employed by de Groot^12^ in the domain of chess. In this task, participants were presented with a chess position for some seconds and were required to reproduce this position afterward. Chase and Simon^13^ extended this task by including both structured and unstructured positions. While structured positions were real game positions, in unstructured positions the pieces were randomly distributed on the board and, thus, meaningless. Only in the structured positions, different levels of expertise were reflected in performance in that stronger players recalled more pieces correctly. In the unstructured positions, in contrast, the performance was largely independent of the expertise level^14^. This pattern of results has been consistently replicated in chess^15^ as well as in other domains of expertise (e.g., music^16^; sports^17^; chemistry^18^; taxi driving^19^) and has been attributed to the experts’ ability to connect the to-be-memorized information with their extensive knowledge base. More specifically, their superior conceptual knowledge would allow experts to recognize familiar patterns and store them parsimoniously in short-term memory^13,20,21^.

Domain-specific knowledge has also been regarded as an important foundation for creative thinking^22,23^. A recent meta-analysis showed that mathematical achievement is positively related to mathematical creativity in children and adolescents (*r* = 0.53^24^) and to a smaller extent in adults (*r* = 0.24^25^). Additionally, mathematical giftedness has been closely associated with mathematical creativity^26^. While there are a few biographical studies on eminent mathematicians^27,28^, where mathematicians emphasized that mathematics is a creative art, that they see mathematics everywhere, and that they are driven by creative avocations, there are no studies yet in which mathematical creativity was measured quantitatively in math experts using well-established creativity tasks. As domain-specific expertise is regarded as highly important for creativity^29^ it can be expected that experts in mathematics, compared to novices, will be more creative in their domain due to their extensive mathematical knowledge. However, it is still unclear if experts are only more creative in their domain or if they are more creative in general. This question leads back to the debate of whether creativity is a domain-specific or a domain-general construct^30,31^. Empirical evidence for both sides can be found, with mathematical creativity sometimes being related to other measures of creativity^25,32–35^, and sometimes not^36–39^. A theoretical model trying to integrate both the domain-specific and the domain-general perspective is the Amusement Park Theory (APT^31,40^). Using an amusement park as a metaphor, creativity is seen as a hierarchical four level model ranging from highly domain-general to highly domain-specific. At the first level are *initial requirements* which are important for creativity in all domains (e.g., intelligence, motivation). At the three subsequent levels, creativity becomes more domain-specific with the underlying traits and skills needed to be creative being highly specific (e.g., *general thematic area*: math/scientific creativity; *domain*: mathematical creativity; *micro domain*: arithmetic creativity). Traits required for creativity are more similar for domains that are within the same general thematic area. Thus, the inconsistent findings regarding the relationship between mathematical and general creativity could derive from a differential reliance on traits and skills on the first (*initial requirements*) and the subsequent levels, which has also been shown by Willemsen et al.^35^ in a large sample of primary school children.

To summarize, research on mathematical expertise is scarce, and very little is known about the interaction between the experts’ extensive knowledge base and information processing. In the present study, we investigate this for memory performance and creativity. In expertise research, short-term memory tasks for domain-specific material are the seminal and among the most frequently employed experimental paradigms for this purpose as they are highly sensitive to individual differences in domain-specific conceptual knowledge. The more elaborate the individual’s conceptual knowledge base is, the more patterns can be identified in the learning material, and the better is the recall even after short presentation times^14,41,42^. So far, there were no studies yet that tested whether experts in mathematics show superior memory performance for structured but not for unstructured material. Also, there is no quantitative research on creativity of experts in mathematics; thus, it remains unclear whether they are only more creative in their domain or in general. This research gap is also addressed in the present study.

We presented a mathematical adaptation of the well-established memory task by Chase and Simon^13^ and mathematical as well as non-mathematical (verbal and figural) creativity tasks to 115 experts in mathematics and to 109 gender, age, and educational level matched novices. For the memory task, in which mathematical information is presented in a structured and unstructured condition, we hypothesized that in the structured condition, experts remember more items than novices. In the unstructured condition, no or a smaller performance advantage of experts is expected. For the creativity tasks, we hypothesized that experts are more creative in mathematics than novices, but it remains unclear whether this is also the case for domain-general creativity.

## Methods

### Participants

In the present online study, we recruited students and faculty members at universities in Austria, Germany, and Switzerland. Approximately 30% from our initial sample (*n* = 349) did not finish the study. We further had to exclude two participants due to insufficient knowledge of German and not having a Bachelor degree. This resulted in our final sample of 224 adults. A sample size of 200 (100 per group) allows the detection of Cohen’s *d* > 0.35 (sensitivity of t-test for independent samples: *p* < 0.050, two-tailed, power = 0.80^43^), thus at least medium effect sizes. Participants were affiliated to one of two groups: Experts (*n* = 115) or novices (*n* = 109). Experts were defined as individuals who have already completed a Bachelor degree (or higher) in mathematics and now study mathematics, and as individuals who currently work at a university in the field of mathematics, either as doctoral candidate, as post-doctoral research fellow or as professor. For novices the same definition was applied but they were from a subject with no to minimal explicit mathematical content (e.g., German Philology, History, Law, Theology, etc.). Both groups were demographically very similar (see Table 1). They did not differ in gender, level of expertise, and nationality. They also did not differ in age, years of domain experience, verbal intelligence, and the self-reported number of hours spent with mathematics until end of (secondary) school. However, the groups differed substantially in their amount of mathematical expertise, indicated by a higher achievement score and almost four times as many hours spent with mathematical activities to the present day. In addition, the experts showed a higher score in figural intelligence. Descriptive statistics, frequentist statistics, and Bayesian statistics for the two groups can be found in Table 2.

**Table 1:** Descriptive statistics, frequentist and Bayesian statistics for experts and novices (Part 1).

Variable	Experts	Novices	Chi square test	BF10	BF01
Gender	65 Male, 48 female, 2 other	48 Male, 60 female, 1 other	Χ^2^(2, 224) = 4.07, *p* = 0.131	0.32	3.13
Level of expertise	62 Master students, 36 PhD students, 17 post-docs/Professors	64 Master students, 37 PhD students, 8 Post-Docs/Professors	Χ^2^(2, 224) = 3.13, *p* = 0.209	0.48	2.09
Nationality	84 Germany, 5 Austria, 19 Switzerland, 7 Other	89 Germany, 9 Austria, 5 Switzerland, 6 Other	Χ^2^(11, 224) = 17.31, *p* = 0.099	2.30e−4	4346.60

**Table 2:** Descriptive statistics, frequentist and Bayesian statistics for experts and novices (Part 2).

Variable	Experts *M* (*SD*)	Novices *M* (*SD*)	Independent samples t-test	Cohen’s d 95% CI	BF10	BF01
Age (years)	27.00 (6.18)	28.47 (5.53)	*t*(222) = − 1.87, *p* = 0.063	*d* = − 0.25 [− 0.51, 0.01]	0.75	1.33
Domain experience (years)	7.34 (6.23)	7.44 (6.00)	*t*(222) = − 0.11, *p* = 0.910	*d* = − 0.02 [− 0.28, 0.25]	0.15	6.81
Verbal intelligence (sum score, 0–20)	13.36 (3.31)	12.97 (3.14)	*t*(218) = 0.89, *p* = 0.374	*d* = 0.12 [− 0.15, 0.39]	0.21	4.68
**Figural intelligence**^**a**^** (sum score, 0–17)**	**7.55 (3.89)**	**4.33 (2.62)**	***t*(189.66) = 7.15, *p* < 0.001**	***d* = 0.97 [0.69, 1.25]**	**5.24e+8**	**1.91e−9**
**Math achievement**^**a**^** (sum score, 0–31)**	**28.23 (2.17)**	**15.96 (6.13)**	***t*(131.40) = 19.55, *p* < 0.001**	***d* = 2.69 [2.32, 3.05]**	**5.24e+46**	**1.36e−47**
**Hours of mathematics**^**a**^	**14,560 (11,834)**	**3963 (11,668)**	***t*(151.98) = 5.62, *p* < 0.001**	***d* = 0.91 [0.57, 1.23]**	**133,294**	**7.50e−6**
Hours of mathematics (until end of school)	3062 (1856)	3627 (11,478)	*t*(161) = − 0.46, *p* = 0.650	*d* = − 0.07 [− 0.38, 0.24]	0.19	5.36
**Hours of mathematics**^**a**^** (after finishing school)**	**10,761 (10,564)**	**206 (466)**	***t*(87.39) = 9.36, *p* < 0.001**	***d* = 1.37 [1.03, 1.71]**	**2.05e+12**	**4.88e−13**

Statistical group differences are highlighted in bold.

^a^Variances of the groups were not equal.

All participants had German as their native language, and reported neither a history of psychiatric, neurological or learning disorders, nor a current use of psychoactive medication. All participants gave written informed consent and were compensated financially. The study has been performed in accordance with the Declaration of Helsinki and was approved by the ethics committee of the University of Graz (07.12.2021/No. GZ. 39/25/63 ex 2021/22).

### Materials

#### Mathematical memory task

The mathematical memory task was designed to be conceptually equivalent to Chase and Simons’^13^ memory task from the domain of chess. Domain-specific material was presented very shortly and needed to be remembered for an evaluation phase. In close collaboration with two mathematicians, we constructed six mathematical items that were each presented in two conditions: the *structured* and *unstructured* condition. In the *structured* condition, the material was structured in a mathematically meaningful way, so that the material should be highly familiar to experts. In the *unstructured* condition, the units of the structured material were presented in a randomized order.

**Figure 1:**
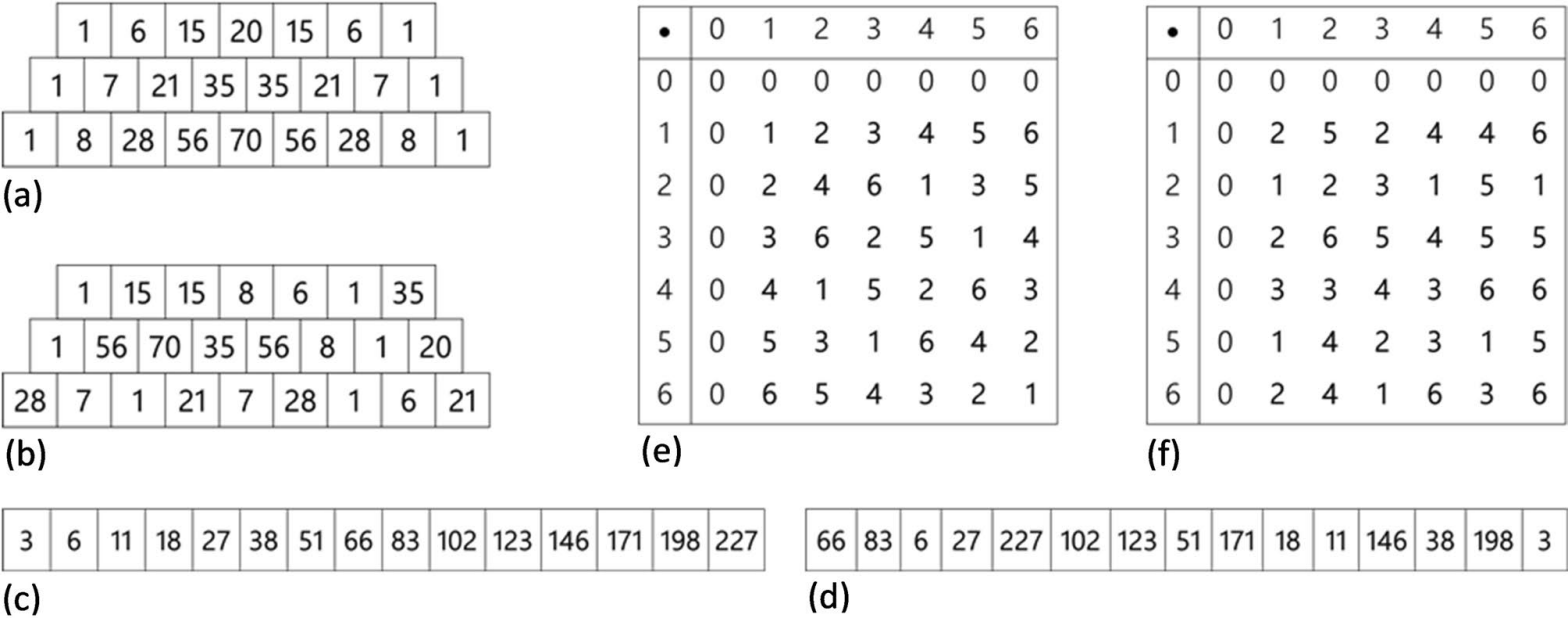
Numerical items of the Mathematical Memory task: *Pascal’s Triangle* (**a**) structured and (**b**) unstructured, *Numerical Series* (**c**) structured and (**d**) unstructured, and *Caylay Table* (**e**) structured and (**f**) unstructured.

The first three items of this task involved numerical material (see Fig. 1). (1) *Pascal’s triangle* In the structured condition we used the sixth, seventh and eighth row of the Pascal’s triangle resulting in 24 numbers. (2) *Numerical series* In the structured condition 15 numbers were generated according to the rule a_n_ = n^2^ + 2 (3) *Cayley table* In the structured condition we used the multiplicative group of integers modulo 7, resulting in 36 numbers. In all three numerical items, the unstructured condition was generated by arranging the numbers of the structured condition randomly. For the analyses we used the sum of correctly recalled numbers at their respective position per item per condition.

**Figure 2:**
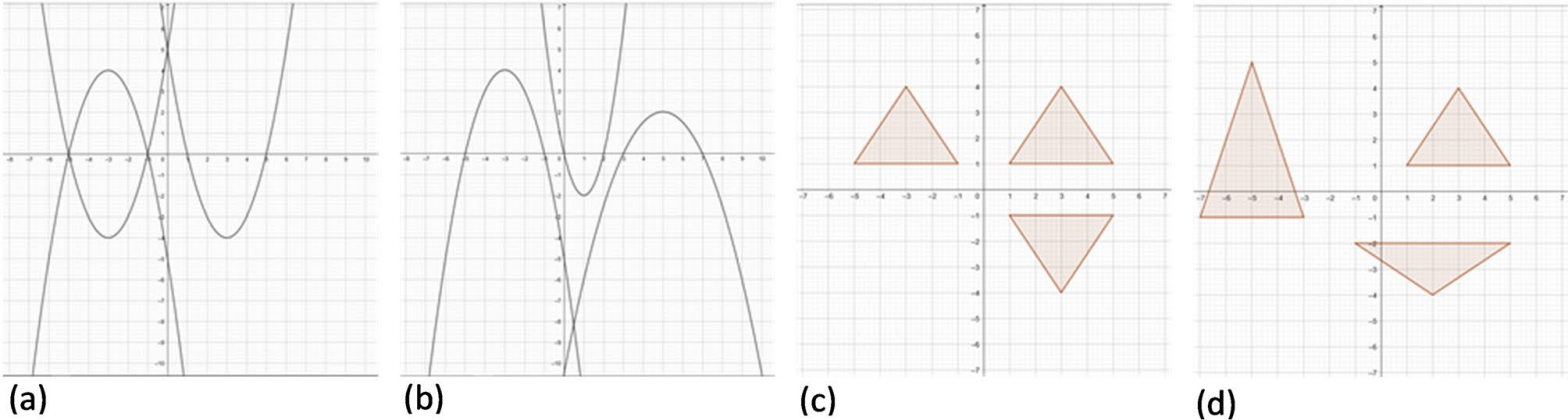
Figural items of the mathematical memory task: *Graphs* (**a**) structured and (**b**) unstructured, *Triangles* (**c**) structured and (**d**) unstructured.

The next two items involved figural material (see Fig. 2). (4) *Graphs* In the structured condition we showed a quadratic function graph reflected across the y- and y-axis in a coordinate system. In the unstructured condition three graphs of quadratic functions, which were not reflecting each other, were presented. (5) *Triangles* In the structured condition we showed an isosceles triangle reflected across the y- and y-axis on a coordinate system. In the unstructured condition we presented three isosceles triangles varying in height which were slightly shifted on the coordinate system. For analyses we manually counted how many of the previously defined points (intersections and vertices for *Graphs* and vertices for *Triangles*) were correctly drawn on the coordinate system. For the *Graphs* item a maximum of 15 points could be reached, for the *Triangles* item, it was a maximum of 9 points.

The last item used verbal material in the form of a theorem with the corresponding proof. (6) *Theorem and Proof* For the structured condition we used the following sentences “Theorem: Suppose n is an integer. If n is even, n^2^ is also even. Proof: If n is even, we can write n as n = 2 k. We then see that n^2^ = (2k)^2^ = 4k^2^ = 2 × 2k^2^. Therefore, n^2^ is even”. In the unstructured condition, we randomized the order of the words. For analyses we manually counted how many words were correctly recalled, with a maximum of 36 points to be reached.

The order of all items was randomized for each participant. After a short instruction to remember the material and which type of material (e.g., numerical, figural, verbal) will be presented, participants were told that they have 20 s to memorize the material and 90 or 180 s, depending on the material, to reproduce the beforehand seen material. The timing was determined by theoretical considerations adhering to the work of Gobet and Simon^42^ and by experimental pilot studies. Overall, participants needed between 15 and 20 min to complete this task. More information about the specific study material and the explicit scoring of the measurements can be found in the Supplementary Information associated with this article.

Considering that the mathematical memory task is a newly created task, we first analyzed its psychometric properties. Due to the dropout rate mentioned above, we had data from more participants than used for the final sample. Thus, we decided to use this larger sample (*N* = 289) to calculate the psychometric properties. All six items of the domain-specific memory task displayed substantial variance and, except for one item, there was no evidence of bottom or ceiling effects. The item that showed a ceiling effect was the structured condition of the *Triangles* item. Here, a maximum of 9 points could be reached, and 252 out of 285 individuals reached the maximum (*M* = 8.51, *SD* = 1.61). Nonetheless, the results suggest that, overall, the mathematical memory had a satisfactory sensitivity. The reliability was evaluated by determining the internal consistency in terms of Cronbach’s alpha. This revealed an acceptable internal consistency (Cronbach’s α = 0.75) across all 12 measurements of the mathematical memory task (structured and unstructured condition of each of the six items). As the structured and unstructured items measure slightly different aspects of memory, we additionally computed the internal consistency for each condition separately. Due to the lower number of measurements, the internal consistency decreased (Structured: Cronbach’s α = 0.59; Unstructured: Cronbach’s α = 0.61). We further looked at convergent and discriminant validity in our final sample and assumed that the overall memory capacity for mathematical material should correlate higher with a related variable than with a dissimilar, unrelated one. To this end, we correlated overall memory capacity in the mathematical memory task with mathematical achievement (*r*(215) = 0.51, *p* < 0.001, 95% CI [0.41, 0.61]) and with verbal intelligence (*r*(217) = 0.06, *p* = 0.359, 95% CI [− 0.07, 0.19]). Indeed, our construct correlated significantly with another mathematical task, but not with a non-mathematical task.

#### Mathematical creativity task

Mathematical creativity was measured with seven items covering three aspects of mathematical creativity: problem-solving, overcoming fixations, and problem-posing^44^. Even though the tasks were originally constructed for children and adolescents, they have been successfully administered in adults^25^.

*Problem-solving* was measured with two figural and two numerical tasks. Before each task, participants were instructed to produce as many solutions as possible to a given mathematical problem within three minutes. *Overcoming fixations* was measured using a figural and a numerical task. In those tasks three items were used to build up a fixation. In the fourth item, a new strategy had to be used to solve the item correctly. Participants were given one point if they overcame this fixation and no point if they gave no answer. *Problem-posing* was measured using one task (adapted from Bicer et al.^45^). In this task, participants were presented with a figure showing in a pictograph how many books were sold per weekday. They had three minutes to make up as many problems as possible and to write down the mathematical formula of the solution.

#### Domain-general creativity

Domain-general creativity was measured with one verbal and one figural task from the Torrance Test of Creative Thinking (TTCT^46^). In the verbal task (*unusual uses*), participants have to find as many unusual uses as possible for a common everyday object, here a tin can. In the figural task (*circles*), participants must draw as many figures as possible from empty circles. We limited the maximum time for each task to 3 min.

All mathematical as well as domain-general creativity items (except for the two overcoming fixation items) were scored for fluency (number of correct answers), flexibility (number of categorically different responses) and originality (judged by five independent raters, divided by fluency to control for fluency bias^47^). For the analyses we z-standardized scores and aggregated fluency, flexibility, and originality for each item. Then we calculated means for each creativity category (problem solving, overcoming fixation, problem posing, verbal creativity, figural creativity), and for mathematical creativity and domain-general creativity in general. More information about the specific study material, scoring and reliability of the measurements can be found in the Supplementary Information associated with this article.

#### Mathematical achievement

Mathematical achievement was measured using the mathematics test for selection of personnel^48^. The official short version consists of 31 mathematical problems and covers topics from higher-order mathematics, namely fractions, conversion of units, exponentiation, division with decimals, algebra, geometry, roots, and logarithm. The maximum processing time was limited to 15 min, but participants could decide to finish earlier. All correct answers were summed up to a raw score ranging from 0 to 31.

#### Intelligence

Intelligence was measured using two tasks from the Intelligence Structure Analysis (ISA^49^). For every item in the verbal task (finding similarities) five words were presented, however, only four of them have something in common. Participants had to find out, which word does not belong to the others. We used the standard set of 20 items and adjusted the time limit to 3 min. In every item of the figural task (recognizing cubes) seven three-dimensional cubes with different patterns on each side are presented. Participants had to find out if the cube presented on the left side is identical to one of the cubes on the right side, and if yes, to which of them. To this end, the cubes had to be mentally rotated. We used the standard set of 17 items and adjusted the time limit to 5 min. All correct answers were summed up to a raw score for each task.

### Procedure

The study consisted of an online test session created with Limesurvey (v. 3.28, http://www.limesurvey.org). In the introduction general information about the study and detailed information about data protection were provided. After participants gave their written informed consent, participants created a unique code to guarantee anonymity. Next, participants reported their demographical data (5 min) and worked through the cognitive tasks. Tasks on mathematical memory (18 min), mathematical creativity (21 min), domain-general creativity (6 min) and mathematical achievement (15 min) were programmed with PsychoPy3 online^50^ and implemented via Pavlovia (https://pavlovia.org/). Each task was initiated via a specific link. Intelligence (10 min) was the last cognitive task and directly implemented in Limesurvey. At the end, participants reported their e-mail address to be contacted for monetary reimbursement. On average, participants needed approximately 75 min to complete the test session.

## Results

### Mathematical memory task

**Figure 3:**
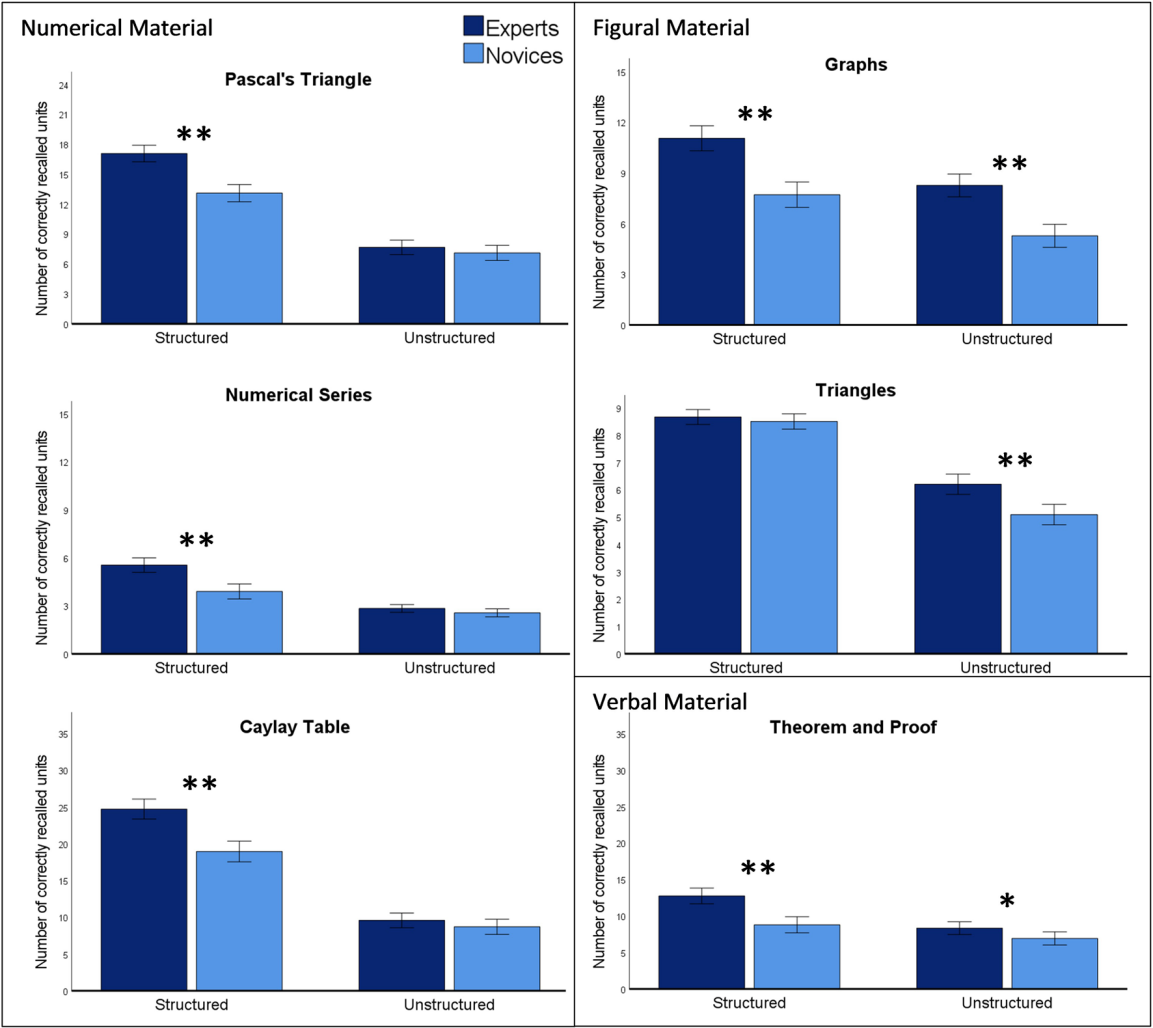
Descriptive statistics for all items of the mathematical memory task; significant differences between groups in the post-hoc tests of the *condition *×* group* are represented by a single asterisk *if *p* < 0.050 and by a double asterisk **if *p* < 0.001; Error bars are 95% Confidence Intervals.

To examine whether experts have a better memory capacity for structured material than novices, we calculated a 2 between-subjects (*group*: experts vs. novices) × 2 within-subjects (*condition*: structured vs. unstructured) mixed ANOVA for each of the six items (see Table 3, Fig. 3). There were no outliers removed and the assumptions of normality and homogeneity of variances were not violated.

**Table 3:** Descriptive statistics for the mathematical memory items separated for experts vs. novices.

Item	Experts—structured *M* (*SD*)	Novices—structured *M* (*SD*)	Experts—unstructured *M* (*SD*)	Novices—unstructured *M* (*SD*)
Pascal’s Triangle	17.07 (4.32)	13.10 (4.76)	7.67 (3.61)	7.11 (4.33)
Numerical Series	5.56 (2.83)	3.91 (1.99)	2.84 (1.30)	2.57 (1.34)
Caylay Table	24.72 (7.99)	18.95 (6.70)	9.58 (5.46)	8.71 (5.38)
Graphs	11.05 (3.71)	7.71 (4.10)	8.26 (3.93)	5.28 (3.15)
Triangles	8.67 (1.44)	8.51 (1.49)	6.09 (1.98)	5.09 (1.98)
Theorem and Proof	12.76 (6.21)	8.80 (5.44)	8.34 (4.35)	6.93 (5.13)

The interaction *group *×* condition* was significant for five of the six items (Pascal’s Triangle: *F*(1, 220) = 23.50, *p* < 0.001, η_p_^2^ = 0.10; Numerical Series: *F*(1, 220) = 13.73, *p* < 0.001, η_p_^2^ = 0.06; Caylay Table: *F*(1, 220) = 20.49, *p* < 0.001, η_p_^2^ = 0.09; Graphs: *F*(1, 212) = 0.46, *p* = 0.501, η_p_^2^ < 0.01; Triangles: *F*(1, 216) = 10.82, *p* = 0.001, η_p_^2^ = 0.05; Theorem and Proof: *F*(1, 221) = 11.84, *p* < 0.001, η_p_^2^ = 0.05). While experts recalled more units than novices in the structured condition of the numerical items (Pascal’s Triangle: Mean Difference (*MD)* = 3.97, *p* < 0.001, *d* = 0.93, BF10 = 1.72e+7, BF01 = 5.83e−8; Numerical Series: *MD* = 1.65, *p* < 0.001 *d* = 0.84, BF10 = 11,501, BF01 = 8.70e−5; Caylay Table: *MD* = 5.77, *p* < 0.001, *d* = 0.89, BF10 = 486,661, BF01 = 2.06e−6), no group difference emerged in the unstructured condition (Pascal’s Triangle: *MD* = 0.56, *p* = 0.297, *d* = 0.13, BF10 = 0.25, BF01 = 4.08; Numerical Series: *MD* = 0.27, *p* = 0.125, *d* = 0.14, BF10 = 0.45, BF01 = 2.23; Caylay Table: *MD* = 0.87, *p* = 0.232, *d* = 0.14, BF10 = 0.29, BF01 = 3.47). In the Triangles item, a group difference emerged in the unstructured condition (*MD* = 1.11, *p* = 0.001, *d* = 0.64, BF10 = 651.58, BF01 = 0.002), but not in the structured condition (*MD* = 0.16, *p* = 0.412, *d* = 0.09, BF10 = 0.20, BF01 = 4.95), probably due to a ceiling effect. Furthermore, experts (compared to novices) recalled more units both in the structured (*MD* = 3.96, *p* < 0.001, *d* = 0.74, BF10 = 15,189, BF01 = 6.58e−5) and the unstructured condition of the Theorem and Proof item (*MD* = 1.41, *p* = 0.027, *d* = 0.27, BF10 = 1.47, BF01 = 0.68), but the difference between groups, indicated by the effect size, was much larger in the structured condition. There was no significant interaction in the Graphs item: experts recalled more units in both the structured (*MD* = 3.34, *p* < 0.001, *d* = 0.89, BF10 = 1.75e+6, BF01 = 5.73e−7) and the unstructured condition (*MD* = 2.98, *p* < 0.001, *d* = 0.80, BF10 = 2.19e+6, BF01 = 4.57e−7).

The overall pattern of the results indicates that, except for the two figural items, the difference in recalled units between experts and novices was larger in the structured than in the unstructured conditions.

The main effect *condition* was significant for all six items (Pascal’s Triangle: *F*(1, 220) = 478.97, *p* < 0.001, η_p_^2^ = 0.69; Numerical Series: *F*(1, 220) = 118.79, *p* < 0.001, η_p_^2^ = 0.35; Caylay Table: *F*(1, 220) = 550.55, *p* < 0.001, η_p_^2^ = 0.71; Graphs: *F*(1, 212) = 97.40, *p* < 0.001, η_p_^2^ = 0.32; Triangles: *F*(1, 216) = 415.30, *p* < 0.001, η_p_^2^ = 0.66; Theorem and Proof: *F*(1, 221) = 71.78, *p* < 0.001, η_p_^2^ = 0.25), confirming that structured material was better remembered than the unstructured material.

Additionally, the main effect *group* was also significant for all six items (Pascal’s Triangle: *F(*1, 220) = 25.02, *p* < 0.001, η_p_^2^ = 0.10; Numerical Series: *F*(1, 220) = 25.83, *p* < 0.001, η_p_^2^ = 0.11; Caylay Table: *F*(1, 220) = 23.67, *p* < 0.001, η_p_^2^ = 0.10; Graphs: *F*(1, 212) = 52.12, *p* < 0.001, η_p_^2^ = 0.20; Triangles: *F*(1, 216) = 11.67, *p* < 0.001, η_p_^2^ = 0.05; Theorem and Proof: *F*(1, 221) = 19.42, *p* < 0.001, η_p_^2^ = 0.08), confirming that experts remembered more units than novices.

### Domain-specific and domain-general creativity

To test whether experts are more creative than novices in mathematical and domain-general (verbal and figural) creativity, we calculated independent t-tests for all items and both tasks (averaged across the respective items). There were no outliers removed and the assumptions of normality and homogeneity of variances were not violated. Experts showed a significant higher performance in all tasks with mathematical material (see Table 4). No significant group differences were found in the domain-general tasks. This pattern was also confirmed by Bayesian statistics. This evidence speaks in favor for substantial group differences in the mathematical tasks and for similarities in the domain-general tasks, supporting our hypothesis that experts are more creative in mathematics than novices.

**Table 4:** Descriptive statistics, frequentist statistics, and Bayesian statistics comparing experts and novices in domain-specific and domain-general creativity items.

Variable	Experts *M* (*SD*)	Novices *M* (*SD*)	Independent samples t-test	Cohen’s d 95% CI	BF10	BF01
**Mathematical creativity**	**0.32 (0.41)**	** − 0.33 (0.40)**	***t*(221) = 11.96, *p* < 0.001**	***d* = 1.60 [1.30, 1.90]**	**7.13e+21**	1.40e−22
**Problem solving**	**0.28 (0.44)**	** − 0.30 (0.49)**	***t*(221) = 9.37, *p* < 0.001**	***d* = 1.26 [0.97, 1.54]**	**3.08e+14**	3.25e−15
**Overcoming fixation**^**a**^	**0.42 (0.84)**	** − 0.44 (0.47)**	***t*(180.31) = 9.51, *p* < 0.001**	***d* = 1.26 [0.97, 1.55]**	**6.13e+14**	1.63e−15
**Problem posing**^**a**^	**0.25 (0.70)**	** − 0.26 (0.79)**	***t*(214.83) = 5.14, *p* < 0.001**	***d* = 0.69 [0.42, 0.96]**	**23,363.13**	4.28e−5
Domain-general creativity	− 0.02 (0.61)	0.02 (0.62)	*t*(219) = − 0.43, *p* = 0.667	*d* = − 0.06 [− 0.32, 0.21]	0.16	6.28
Verbal creativity	− 0.02 (0.72)	0.02 (0.84)	*t*(219) = − 0.36, *p* = 0.718	*d* = − 0.05 [− 0.31, 0.22]	0.15	6.70
Figural creativity	− 0.02 (0.93)	0.02 (0.67)	*t*(219) = − 0.31, *p* = 0.760	*d* = − 0.04 [− 0.31, 0.22]	0.16	6.13

Variables, where the t-test showed significant group differences after Bonferroni correction for multiple comparison (α = 0.050/7 → α = 0.007) are bolded; all scores are mean z-scores.

^a^Indicates that the variances of the groups were not equal. BF01 represents evidence for the null hypothesis (no difference between groups); BF10 represents evidence for the alternative hypothesis (difference between groups). BFs bigger than 1 indicate anecdotal evidence, BFs bigger than 3 provide moderate evidence, BFs bigger than 10 provide strong evidence 30.

## Discussion

The aim of the present study was to investigate the interaction between expert knowledge in mathematics and domain-specific information processing in memory as well as domain-specific and domain-general creativity. Using a mathematical adaptation of Chase and Simon’s^13^ pioneering memory task, we demonstrated that experts (compared to novices) have a higher memory capacity for structured mathematical material but not or to a smaller extent for unstructured, mathematically meaningless material. In addition, our results in well-established creativity tasks revealed a higher performance of experts only in domain-specific (mathematical) but not in domain-general (verbal and figural) creativity tasks.

The interactions between group and condition in the mathematical memory task revealed the expected pattern of experts outperforming novices only in the structured condition in three of the six memory items, all containing numerical material (i.e., *Pascal’s triangle, Numerical Series, and Caylay Table*). In the verbal item (*Theorem and Proof*), the group difference in favor of experts was larger in the structured than in the unstructured condition. These results are in line with our hypothesis as well as with previous work in several domains^13,16–19^, and indicate that experts were successful in recognizing the underlying mathematical structure due to their large and well-organized domain-specific knowledge base. The items differed with regard to how helpful identifying the underlying structure was. For example, if one was able to identify the underlying structure of the *Pascal’s Triangle*, it was possible to reproduce it by using the appropriate procedural strategy. In contrast, for the *Theorem and Proof,* identifying the underlying structure (mathematically correct theorem and the corresponding proof) was beneficial for remembering but not sufficient to reproduce all units/words. In the latter item, the small group difference in the unstructured condition may have occurred because experts perceived some meaningful patterns by chance^14^.

In contrast to our hypothesis, we did not observe an expert advantage only for structured information in the figural items. In the *Graphs* item, experts showed better memory recall in both the structured and unstructured condition. In the *Triangles*, a group difference in favor of experts emerged only in the unstructured condition. The group difference in the unstructured condition may be due to the experts’ larger familiarity with the learning material and the associated perceptual advantage^1,51^. Specifically, in contrast to numerical information that is part of everyday life, the coordinate system in the figural items may be less familiar to novices than to experts. Consequently, because of their knowledge, experts may have automatically focused their attention on the relevant information (intersections and vertices), whereas novices may have tried to store the entire figure. This information processing difference could explain the similarly strong effect of expertise in the structured as well as unstructured condition of the *Graphs* item. Further, experts had higher figural intelligence scores than novices, which could allow them to manipulate figural information more easily in general. The lack of group difference in the structured condition in the *Triangles* item, in contrast, can very likely be traced back to a ceiling effect.

Another finding shown by the post-hoc effects is that novices remembered more units in all structured compared to unstructured conditions. This stands in contrast to findings from other domains, where novices performed similarly in both conditions^16,19,52^. This divergence is very likely due to the expertise domain and the definition of novices. Even though the novices received no tertiary education in mathematics, they had a considerable amount of mathematical knowledge acquired in math education during primary and secondary school, while in the studies reported above, novices had no or minimal experience with the task material. The presentation time of 20 s was probably long enough for novices to also discover some of the underlying structures within our experimental materials.

Our results in the creativity tasks revealed that experts exhibited higher performance in domain-specific (mathematical) but not domain-general (verbal, figural) creativity. The superiority of experts in domain-specific creativity replicates the findings from other expertise domains (e.g., music^53^) and corresponds to results in non-experts showing that higher mathematical expertise is related to higher mathematical creativity^25,54^. They do not support the assumption that experts have more rigid thoughts, which hinders them to be creative^1,55^; rather, prior knowledge and experiences can be regarded as the basis for creative thinking^23^. Further, the present results suggest that mathematical creativity strongly relies on domain-specific skills. According to the APT^31,40^, mathematical and domain-general creativity fall into different *general thematic areas*, thus not requiring the same traits to be creative. This is further supported by a non-significant correlation of mathematical creativity with domain-general creativity (*r* = 0.13, *p* = 0.056). To some extent, the present findings correspond to the recent study by Palmiero et al.^53^ who reported a significant difference between expert musicians and self-taught musicians/non-musicians in domain-general verbal creativity but not in domain-general visual creativity. The authors explained this result as the consequence of shared musical and verbal processing mechanisms. While this is a plausible explanation, there is also another methodological possibility. Since the groups were not matched for intelligence, the differences could have also been driven by differences in general verbal intelligence, as according to the APT, intelligence is an *initial requirement* for creativity in all domains^40^. However, in the present study, experts and novices did not differ in verbal intelligence, even though the experts had a higher figural intelligence score than novices. Thus, this could be one reason why we did not observe group differences in our measures of domain-general creativity. Future research needs to clarify whether some of the reported associations between domain-general creativity and expertise are genuine effects or methodological artifacts.

A first limitation of the present study lies in the online administration of the tasks. While this procedure allowed us to investigate the up-to-date largest sample of experts in mathematics, the environment of the participants in the test session may have varied. Parameters influencing the performance like time of day, a quiet environment, and interruptions could not be controlled. Further, we have no insight into the participants’ commitment, even though one could argue that all participants who spent approximately 75 min on cognitively demanding tasks had to show at least some amount of engagement. Second, while both the mathematical memory as well as the mathematical creativity task could successfully differentiate between experts and novices, we do not know to what extent it can differentiate within experts. Further studies, using more complex items for both memory and creativity, should explore this question, especially as previous studies could show that even within experts differences occur in memory^42^ and mathematical creativity^56^. Third, while mathematical memory might be one concept to investigate the interaction between the exceptional mathematical knowledge base of experts and information processing, future studies should also focus on other promising concepts (e.g., a mathematical cast of mind, attentional biases towards numerical information).

In conclusion, using a novel mathematical memory task, we could demonstrate that experts have a higher memory capacity for complex domain-specific material when it is representative of their domain of expertise. To some extent, this performance advantage generalizes to unstructured material in which the experts still may be able to benefit from their vast mathematical knowledge during encoding and retrieval. Experts also exhibited a higher mathematical creativity than novices but did not differ from them in their domain-general creativity, providing further evidence for a domain-specific perspective of creativity. Both lines of findings corroborate the importance of the experts’ knowledge base in the perception and processing of domain-specific material and provide new insights into the characteristics of mathematical expertise.

## Supplementary Information

Supplementary Information.

## Data Availability

The data that support the findings of this study along with the data-analysis scripts are posted at OSF https://osf.io/xd7kv/?view_only=b73ebe4bbb1a410fbea1108705305e9c. More information about the materials used can be found in the Supplemental Online Material. The manuscript was uploaded as preprint on 20.09.2022 at PsyArXiv under CC-By Attribution 4.0 International and can be found under 10.31234/osf.io/wvqtn.
