# Supplementary material for: Mathematical expertise: the role of domain-specific knowledge for memory and creativity
Source: Sci Rep. 2023 Aug 2;13:12500. doi: 10.1038/s41598-023-39309-w (PMC10397219; doi:10.1038/s41598-023-39309-w)
Supplement: Supplementary file 1 — Supplementary Information. [file 41598_2023_39309_MOESM1_ESM.pdf]

Mathematical expertise: The role of domain-specific knowledge for memory and creativity

Michaela A. Meier[1], Franz Gross[1], Stephan E. Vogel[1] & Roland H. Grabner[1]*

[1]*University of Graz, Austria*

*Corresponding author: Universitätsplatz 2, 8010 Graz, Austria, +43 316 380 – 8473, michaela.meier@uni-graz.at

Supplementary information

1. Mathematical structure in the Mathematical Memory items

The first three items of this task involved numerical material. (1) *Pascal's triangle*. The Pascal's triangle starts with the number 1 in the top row and the row below is constructed by summing the two adjacent elements in the preceding row (as such the triangular array can be expanded infinitely). In the structured condition we used the sixth, seventh and eighth row of Pascal's triangle resulting in 24 numbers. In the unstructured condition, the 24 numbers were arranged randomly. (2) *Numerical series*. In the structured condition numbers were generated according to the rule an = n^2+2. The number series started with n=1 and therefore with the number three and the following 14 numbers in this sequence were calculated resulting in 15 numbers to remember. In the unstructured condition the numbers were arranged randomly. (3) *Cayley table*. A Cayley table is a technique for describing an algebraic structure (usually a finite group) by putting all the products of the group's elements in a square array. For the structured condition we used the multiplicative group of integers modulo 7. The respective Cayley table contained 36 numbers. In the unstructured condition the numbers were arranged randomly. For the analyses we used the sum of correctly recalled numbers at their respective position per item per condition.

The next two items involved figural material. (4) *Graphs*. In the structured condition we showed three graphs of quadratic functions in a coordinate system. The graph of the function f(x) = (x - 3)2 – 4, which was reflected across the y-axis resulting in the graph of the function g(x) = (-x - 3)2 – 4, which in turn was reflected across the x-axis resulting in the graph of the function h(x) = -((-x - 3)2 – 4). In the unstructured condition three graphs of quadratic functions, which were not reflecting each other, were presented. (5) *Triangles*. In the structured condition we showed three isosceles triangles on a coordinate system. Again, one triangle was reflected across the x-axis and across the y-axis. In the unstructured condition we presented three isosceles triangles varying in height which were slightly

shifted on the coordinate system. For analyses we manually counted how many of the previously defined points (intersections and vertices for Graphs and vertices for Triangles) were correctly drawn on the coordinate system. For the Graphs item a maximum of 15 points could be reached, for the Triangles item, it was a maximum of 9 points.

The last item used verbal material in the form of a theorem with the corresponding proof. (6) *Theorem and Proof*. For the structured condition we used the following sentences "Theorem: Suppose n is an integer. If n is even, n2 is also even. Proof: If n is even, we can write n as n = 2k. We then see that n2 = (2k)2 = 4k2 = 2 x 2k2. Therefore, n2 is even.". In the unstructured condition, we randomized the order of the words but kept the position of theorem and proof and the length of both approximately the same. Formulas were not separated when randomizing the order of the words. For analyses we manually counted how many words were correctly recalled, with a maximum of 36 points to be reached.

2. Explicit scoring scheme of the Mathematical Memory task

While the three items, which used numerical material, were scored automatically using a Python script, the two figural and the one verbal task were scored manually.

For the two figural items we manually counted how many of the previously defined points (intersections and vertices for Graphs and vertices for Triangles) were correctly drawn on the coordinate system. We decided give one point per perfectly recalled unit, but also give 0.5 points if for example in the triangle item, participants drew a line over the vertex (e.g., (1, 1)), but their vertex was at a neighboring location (e.g., (1, 0)). Further, participants had to draw the shapes they remembered using the mouse, so often they might remember the intersections or vertices correctly, but drew them slightly offset, due to limits of usability of this task. Thus, it was up to the scoring person to decide which amount of deviation still counted as correct. Due to this room for individual scoring, we decided to use three independent raters to score the two figural items. To assess objectivity in scoring we calculated the ICC at response level, which was excellent for all items (after Koo & Li[1]; Graphs - Structured: ICC = .98; Graphs - Unstructured: ICC = .99; Triangles - Structured: ICC = .99; Triangles - Unstructured: ICC = .99). For all further analyses, a mean score of the three raters was used. For the Graphs item a maximum of 15 points could be reached, for the Triangles item, it was a maximum of 9 points.

For the verbal item we manually counted how many words were correctly recalled. We decided to give one point per perfectly recalled word, but also give 0.5 points if participants dropped one word (e.g., "Theorem:") but all the other following words were correct again. In the latter case, strictly scored, all the following words would be incorrect, because they were not in the correct absolute position anymore. Due to this room for individual scoring, we decided to use three

independent raters also for the verbal item. Again, objectivity was excellent for all items (Theorem and Proof - Structured: ICC = .99; Theorem and Proof - Unstructured: ICC = .99). For all further analyses, a mean score of the three raters was used, and a maximum of 36 points could be reached.

3. Mathematical creativity items

The *problem-solving* items were the following: (1) *Figural Generate*[3]. Participants had to form shapes with a size of exactly 2 cm² within given 4 cm² squares (represented by nine points with a respective distance of 1 cm) by connecting the points with lines. (2) *Figural Similarities*[4]. Eight different geometric figures (figure A–figure H) were shown, in which common properties between figure B and one or more of the other figures had to be found. (3) *Numerical Similarities*. This task was adapted from Haylock[5] and used in Meier et al.[6]. Participants had to identify as many similarities as possible between the numbers 16 and 36. (4) *Numerical Generate*[7]. Participants had to fill in the blanks on the two sides of an equal sign by using the digits 1, 2, 3, 4, 5, 6 and the mathematical symbols (+,-,×,÷,(,)) in order to create an equality.

Overcoming fixations items were the following: (5) *Cuts*[5]. Participants were asked to divide a rectangle into a given number of equal parts. In the example item, the rectangle can be cut into two equal parts using one vertical line. For the next three items, participants had to give the answer for three, five, and seven parts, and had a maximum of 45 seconds for each item. The correct answer is always the number of parts minus one. In the fourth item in this task, participants were asked to divide the rectangle in nine parts. Even though eight vertical lines is a correct answer, the more creative answer, where the algorithmic fixation is broken, would be two horizontal and two vertical lines. (6) *Sum and difference*[5]. Participants had to find the two numbers with a given sum and difference. The example with a sum of ten and a difference of four had the answer seven and three and lead the participants to think in the content-universe of positive integers. The next three items (sum 12 & difference 4; sum 7 & difference 3; sum 8 & difference 4) enforced this fixation, and participants again had a maximum of 45 seconds for each item. In the fourth item in this task, the participants were asked to find the two numbers with a sum of nine and a difference of two. Here the correct answer was 3.5 and 5.5, therefore participants had to overcome the self-restriction on whole numbers as answer. In both overcoming fixation tasks, participants were given one point if they overcame this fixation and no point if they gave no answer (or the algorithmic solution in Cuts task).

The Problem-posing item was the following: In this task, originally from Bicer et al.[8], participants were presented with a figure showing in a pictographic way how many books were sold per weekday. Using this figure, they had three minutes to make up as many problems as possible. Next to the mathematical problem, they also had to write down the mathematical formula of the solution.

4. Explicit scoring scheme of mathematical creativity and domain-general creativity

All mathematical as well as domain-general creativity items (except for the two overcoming fixation items) were scored for fluency, flexibility and originality.

Fluency was operationalized as the number of correct answers. Flexibility was operationalized as the number of categorically different responses. Originality was judged by 5 independent raters. We decided to not rate the originality of the domain-general creativity items based on the manual of the TTCT [9] to be consistent with the scoring of the mathematical creativity test, as well as for limitations in the original scoring method. The inter-rater reliability, evaluated through the intraclass correlation coefficient (ICC) at response level, was moderate or good for all tasks (Figural Generate: ICC = .87; Figural Similarities: ICC = .69; Numerical Similarities: ICC = .84; Numerical Generate: ICC = .87; Problem Posing: ICC = .88; Unusual uses: ICC = .55; circles: ICC = .72). Cronbach's α as measure for internal consistency of the measurement was good (if fluency, flexibility, and originality are entered as separate scores Cronbach's α = .82, if aggregated scores per item are entered Cronbach's α = .71) indicating one underlying construct. For further analyses we first divided the summed-up originality score through the fluency score to reduce the confounding effect of fluency [10]. Second, we z-standardized each score to allow the building of unweighted composite cores. Third, we computed mean scores for each creativity item. Fourth, we averaged the items on a higher level leading to one score for each creativity category (problem solving, overcoming fixation, problem posing, verbal creativity, figural creativity), and averaged those to a mathematical creativity score and a domain-general creativity score.

1. Koo, T. K. & Li, M. Y. A Guideline of Selecting and Reporting Intraclass Correlation Coefficients for Reliability Research. *J. Chiropr. Med.* **15**, 155–163 (2016).
2. Sala, G. & Gobet, F. Experts' memory superiority for domain-specific random material generalizes across fields of expertise: A meta-analysis. *Mem. Cogn.* **45**, 183–193 (2017).
3. Haylock, D. W. A framework for assessing mathematical creativity in school chilren. *Educ. Stud. Math.* **18**, 59–74 (1987).
4. Becker, J. P. & Shimada, S. *The Open-Ended Approach: A New Proposal for Teaching Mathematics.* (National Council of Teachers of Mathematics, 1997).
5. Haylock, D. Recognising mathematical creativity in schoolchildren. *ZDM - Math. Educ.* **29**, 68–74 (1997).
6. Meier, M. A., Burgstaller, J. A., Benedek, M., Vogel, S. E. & Grabner, R. H. Mathematical Creativity in Adults: Its Measurement and Its Relation to Intelligence, Mathematical Competence and General Creativity. *J. Intell.* **9**, 10 (2021).
7. Kontoyianni, K., Kattou, M., Pitta-Pantazi, D. & Christou, C. Integrating mathematical abilities and creativity in the assessment of mathematical giftedness. *Psychol. Test Assess. Model.* **55**, 289–315 (2013).
8. Bicer, A., Lee, Y., Perihan, C., Capraro, M. M. & Capraro, R. M. Considering mathematical creative self-efficacy with problem posing as a measure of mathematical creativity. *Educ. Stud. Math.* **105**, 457–485 (2020).
9. Torrance, E. P. Torrance Tests of Creative Thinking: Directions Manual and Scoring Guide. at (1966).
10. Forthmann, B., Jankowska, D. M. & Karwowski, M. How reliable and valid are frequency-based originality scores? Evidence from a sample of children and adolescents. *Think. Ski. Creat.* **41**, 100851 (2021).
